# Gender bias and menstrual blood in stem cell research: A review of pubmed articles (2008–2020)

**DOI:** 10.3389/fgene.2022.957164

**Published:** 2022-12-01

**Authors:** Daniela Tonelli Manica, Karina Dutra Asensi, Gaia Mazzarelli, Bernardo Tura, Germana Barata, Regina Coeli Santos Goldenberg

**Affiliations:** ^1^ Laboratory of Advanced Studies in Journalism, State University of Campinas, Campinas, Brazil; ^2^ Carlos Chagas Filho Institute of Biophysics, Federal University of Rio de Janeiro, Rio de Janeiro, Brazil; ^3^ Institute of Earth Sciences, State University of Campinas, Campinas, Brazil; ^4^ National Institute of Cardiology, Research and Teaching Department Rio de Janeiro, Rio de Janeiro, Brazil; ^5^ National Institute of Science and Technology for Regenerative Medicine—REGENERA, Federal University of Rio de Janeiro, Rio de Janeiro, Brazil

**Keywords:** mesenchymal stromal cells, stem cells, menstrual blood, gender, research policies, altmetrics, pubmed, science communication

## Abstract

Despite proven scientific quality of menstrual blood mesenchymal cells, research and science output using those cells is still incipient, which suggests there is a resistance to the study of this type of cell by scientists, and a lack of attention to its potential for cell therapy, regenerative medicine and bioengineering. This study analyzes the literature about the menstrual blood mesenchymal stromal/stem cells (mbMSC) on the PubMed database between 2008–2020 and the social attention it received on Twitter. A comparative analysis showed that mbMSC accounts for a very small portion of mesenchymal cell research (0.25%). Most first authors are women (53.2%), whereas most last authors are men (63.74%), reinforcing an already known, and still significant, gender gap between last and corresponding authors. Menstrual blood tends to be less used in experiments and its scientific value tends to be underestimated, which brings gender bias to a technical and molecular level. Although women are more positive in the mbMSC debate on Twitter, communication efforts toward visibility and public interest in menstrual cells has room to grow.

## Introduction

In this article, we look at recent (2008–2020) scientific papers on menstrual blood mesenchymal stromal cells. We searched the PubMed database to compare publications with research results about these cells with studies about other types of mesenchymal cells used in the biological sciences. Our goal was to evaluate the representativeness of this research in the field.

We analyzed the sociological profile of the authors of these articles, taking into consideration mainly the gender and country of the first and last authors. A second objective was to look at their repercussions on social networks, focusing on Twitter, and using complementary metrics of social attention (altmetrics). We surveyed the profiles of users who published posts disseminating and/or commenting on the articles about menstrual blood cells on the network in terms of gender (whether they were male, female, or non-genuine group profiles) and the tone of the comments (positive, negative, neutral).

The aim of the study was to observe the gender dynamics that conform scientific practices, trying to think what place is given to women and their bodies in the scientific research agenda in the field and in their Twitter communications. Contrary to popular belief, science is not neutral in its choices of research objects, nor in its institutional dynamics. Taking that in consideration, we hope to contribute to the development of an ethical, equitable and diverse science, able to neutralize gender and race biases that amplify social inequalities.

Studies in the last half century have broadly considered gender issues in science (Keller, 1985; Harding, 1986; Fausto-Sterling, 1992; Keller and Longino, 1996; Haraway, 1997; Schiebinger, 1999; Fausto-Sterling, 2000; Allagnat et al., 2017). Feminist and postcolonial approaches to science have recently shown the complex interrelations between inequalities of gender, race/ethnicity, and class within scientific practices (Benjamin, 2013; Roy, 2018). The presence of gender and racial bias in science is well-known, and many studies focus on the presence and proportion of women and other minorities in different social contexts within laboratories and universities.

Recent efforts, at times associated to the political agendas of social movements, have sought to increase the visibility of the common social dynamics that limit the scientific careers of women and other minorities. Reports show that women tend to have lower remuneration and peer acknowledgment, and that there is a bottleneck, or a glass ceiling, for women and other minorities, impeding them from achieving higher status in academic and other power hierarchies (Woolston, 2019). Demands over women’s evaluation tend to be higher (Hengel, 2017), and experiments with non-blind reviews have shown that they tend to favor white men (Budden et al., 2008). Male authors may be associated with greater scientific quality (Knobloch-Westerwick et al., 2013) and gender bias influences the review process (West et al., 2013). Furthermore, gender and international diversity are related to lower acceptance rates in peer reviews (Murray et al., 2019).

In November 2020, the publication of a paper by Nature Communications shook the scientific community (AlShebli et al., 2020). Based on an analysis of shared authorship in papers and citation rates, the authors concluded that “male” mentors and protégées had more benefits than “females”. The paper had to be retracted after receiving criticism about what counted as “informal mentorship”, and because it only considered the metrics of citation rates and their impact on evaluating careers in sciences. The authors were accused of leaving aside important gender disparity issues like underrepresentation; the smaller access of women to grants and leadership roles, demonstrated in numerous studies and reports (Allagnat et al., 2017; Royal Society of Chemistry, 2018; Kleijn et al., 2020); and the salary gender gap present even in science (Woolston, 2019). The authors “did not acknowledge their unjustified conclusions relating female gender to career success and policy suggestions” (Mummery et al., 2020).

Publishers and academic associations have been forced to review their gender and racial biases, and publication and admission policies (Wu, 2020). Racism and sexual harassment events came to be addressed institutionally. In recent years, many journals and scientific associations have committed to reducing inequalities in publishing procedures and towards the equality of gender and the inclusion of diversities (Heidari et al., 2016; IOP Publishing, 2018; Royal Society of Chemistry, 2018; Beeler et al., 2019; Dyer et al., 2019) among others. Bringing these situations to the public and producing data about the inequalities made publishing policies more explicit, and made inclusion and diversity into important topics to be considered for good scientific practices. But we still have a long way to go “to make significant changes for gender equality” (Mummery et al., 2020).

Gender studies have contributed largely to legitimizing discussions about equity and representation in many areas of social life, such as science and technology. But “the question of science in feminism” (Harding, 1986) has also led to analyses that situate scientific discourse and practices as socially, culturally, and politically marked (Martin, 1987; Haraway, 1997; Barad, 2007). In that sense, the very concept of “sex” or “sexual differences” became historically and culturally situated, shifting criteria for what counts as “female” and “male” from a given-natural-material-bodily difference to processes that are more open, variable, complex, and co-produced (Jasanoff, 2004). The way each culture and society defines “female” and “male”, “women” and “men” vary greatly (Strathern, 1988).

Menstruation and menstrual blood are tied within this complexity (Bobel, 2010; Bobel et al., 2020), being mostly associated to “female” bodies, bodies understood as feminine and of cis women–although trans men, non-binary people, women in menopause and women without a uterus complicate this association. We cannot limit the relations to menstruation and gender identities. In this study, we assume menstrual blood is a bodily fluid that socially and culturally marks differences between women and men, being understood as a specific phenomenon that happens to most cis women during a long period of their lives. We will consider its presence in scientific laboratories, relating to mesenchymal stromal/stem cells and to the scientists that work with them in their research.

As a recent meeting report shows, “women and women’s health concerns” is “underrepresented in research” (Critchley et al., 2020), in spite of all the efforts to reduce the gender inequalities in clinical studies conducted in the United States of America. The authors show that, of the more than 1000 mesenchymal stem cell clinical trials listed on ClinicalTrials.gov in 2018, “only 2 use menstrual blood derived cells, both taking place at Zhejiang University in Hangzhou, China: 1 for chronic liver disease and 1 for type 1 diabetes” (Critchley et al., 2020). Departing from the perception of the fact that menstrual blood seems to be “surprisingly understudied” (Critchley et al., 2020), yet of high quality to stem cells research, and considering gender inequalities in science, we ask the following questions.

What can we tell about the relations between menstrual blood and gender when we look at research on menstrual blood mesenchymal stromal/stem cells (mbMSCs)? How are microscopes, scientific papers, journals, social networks, and power relations between women and men, seniors and juniors, from the global North and South articulated to co-produce differences related to gender and science? To address these questions, we will focus on results from mbMSC research.

In the 1970s, Friedenstein and colleagues were the first to report the existence of a type of adult stem cell in the bone marrow stroma, different from hematopoietic stem cells. These cells were clonogenic, adherent to the culture flask, of a fibroblastic shape (Friedenstein et al., 1970; Friedenstein et al., 1974), and named as mesenchymal stem cells (MSCs) (Caplan, 1991). Since then, these cells have been extensively studied and their main mechanism of action has been described through paracrine secretion (Caplan and Dennis, 2006; Caplan and Correa, 2011). These cell-derived products, such as extracellular vesicles, trophic and immunomodulatory factors, have shown significant benefits *in vivo* (Nagaishi et al., 2016; Xia et al., 2019; Zhang et al., 2020; Santamaria et al., 2021). Considering the growing publications demonstrating the beneficial paracrine effect promoted by the released factors, another proposed name for them was “Medicinal Signaling Cells” (Caplan, 2010; Caplan, 2017).

The MSCs are found in a variety of tissues in the human body and in extra-embryonic attachments and are characterized according to the minimal criteria established by the International Society for Cell Therapy (Williamson and Minter, 2019). However, the extraction of many of these MSCs needs invasive procedures, such as bone marrow and adipose MSCs. The MSC derived from extra-embryonic attachments are also only available once, at birth, or by invasive procedures during pregnancy, such as amniocentesis. In this context, menstrual blood is a unique MSC source, available monthly throughout the reproductive lives of cis women, and safely collected without invasive procedures by the donor (Critchley et al., 2020). But we will demonstrate it is one of the least studied sources of MSCs despite all these advantages of working with mbMSC, such as their availability for decades throughout women’s lives and can be easily and painlessly collected.

We analyzed the presence and prevalence of mbMSCs in scientific papers involving mesenchymal cells published in 12 years (2008–2020) and included in the Pubmed database. We compared the uses of mbMSCs with other mesenchymal stromal/stem cells also being studied (like bone marrow, umbilical cord, adipose tissue, placenta, dental pulp, amniotic fluid, and endometrium).

Our focus is on the relation between gender issues and the presence or absence of mbMSC in the group of bodily tissues usually employed in the fields of regenerative medicine, bioengineering, and cell therapy. How many of the papers published in these 12 years about mesenchymal stromal/stem cells used menstrual blood as a source? Who were the scientists involved in these studies with menstrual blood in terms of gender? In which countries are their institutions located? Are the results similar to the ones found in mainstream publications in the field of stem cells? What is the impact factor of the journals that published research with mbMSCs? Are there similarities to most publications in the field? What is the social attention received by mbMSCs on Twitter? What could be improved in the communication of mbMSCs?

Our hypothesis is that menstrual blood is undervalued in mesenchymal stromal/stem cell research, and that women scientists are the ones mostly involved in research using it. We also expect to see that research related to menstrual blood stem cells lacks social attention on Twitter, the attention it gets is primarily by women, and tweets may reveal bias against the use of mbMSC.

## Materials and methods

**FIGURE 1:**
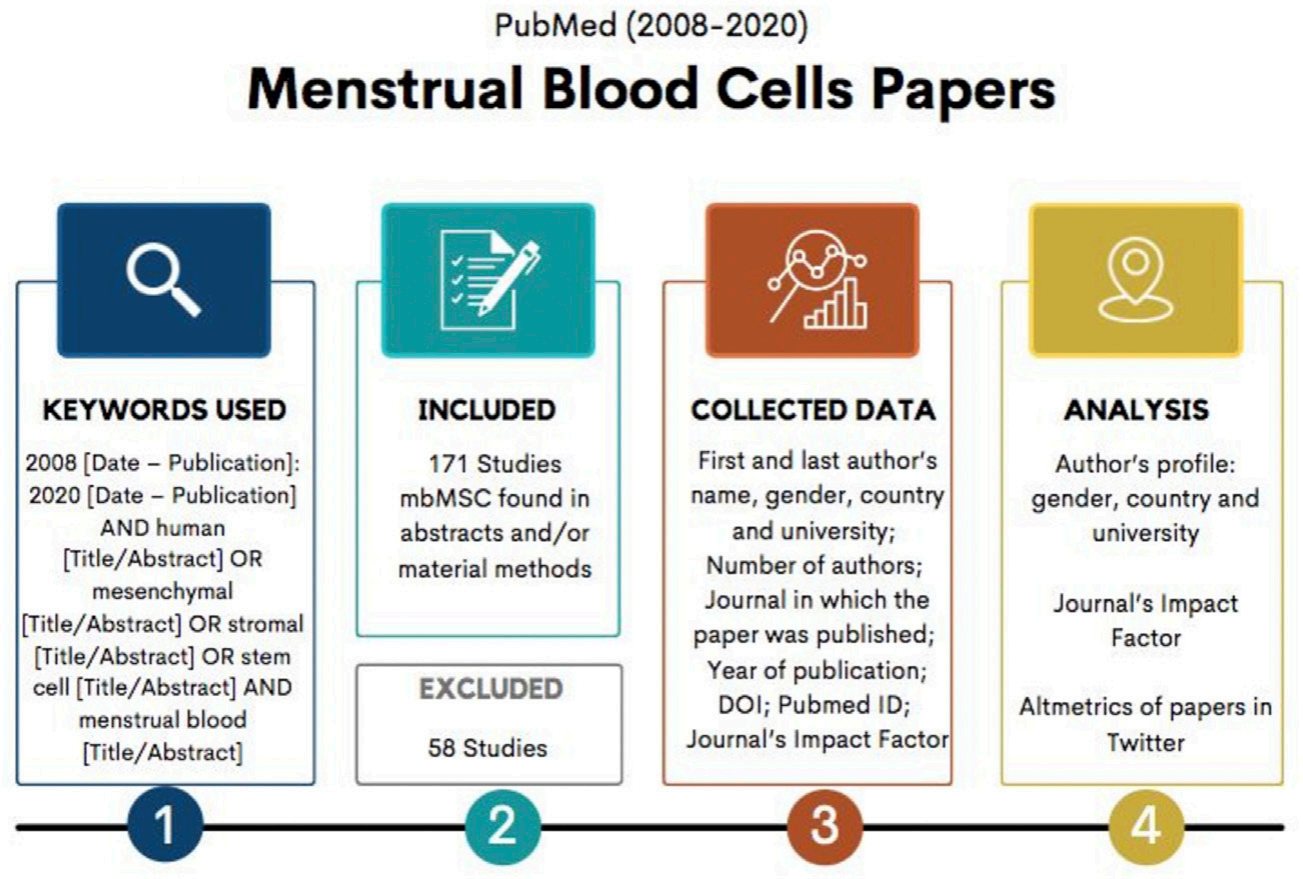
Flowchart with research methodology.

Our analysis is focused on research about mesenchymal stromal/stem cells in the PubMed database. Pubmed is a publicly available repository for medical literature and probably the main source for medical literature (Williamson and Minter, 2019) and, therefore, relevant to scientific research results on stem cells. Flowchart of the research methodology is illustrated in Figure 1.

Considering the first studies about menstrual blood mesenchymal stromal/stem cells were published in 2007, we took 2008 as initial date for our search and applied a 12-year period (2008–2020). The following terms and keywords were used: 2008 (Date–Publication): 2020 (Date–Publication) AND human (Title/Abstract) OR mesenchymal (Title/Abstract) OR stromal (Title/Abstract) OR stem cell (Title/Abstract) AND menstrual blood (Title/Abstract). A total of 229 results were found. All abstracts were individually read, and, in most of the publications, the item “materials and methods” too. Research that did not use menstrual blood as a source for mesenchymal cells was discarded. We have then selected 171 articles for the analysis.

The 171 articles were downloaded and tabulated with the following information: first author’s name, gender, country and university; last author’s name, gender, country and university; number of authors; journal in which the paper was published; year of publication; DOI; Pubmed ID; journal’s Impact Factor. The results allowed us to analyze the author’s profile, comparing gender, country and university, the journal’s Impact Factor, as well as the articles’ altmetrics in Twitter. We also mapped the flux of publications throughout the 12 years.

We considered “gender” as the social expression of a difference understood by western culture as “sexual”, and as something that can be represented by the person’s name and physical appearance. Genders of first and last authors were compared, along with the profiles of countries and universities. We aim at addressing the various scales or levels in which gender dynamics are involved: considering how women and men are present among these scientists and how cells, such as menstrual blood cells, come to be gendered and what that implies in terms of scientific results and impacts.

Gender assignment was made by inference using the first name and the profiles in universities’ websites, in academic social networks (academia.edu and researchgate.net), and in private social networks (Facebook and Twitter). Ambiguous names, especially of researchers from China and Japan, whose names do not have a gendered written form in Roman alphabet, were analyzed by using the software genderize. io. We have only considered the results with 60% or higher probability of being right. Cases of non-binary and trans people were disregarded in this analysis. The data will be presented with absolute and relative frequencies, and to evaluate the differences between the groups the chi-square test or Fisher’s exact test were used. The tests were performed with R version 4.0 software.

To compare the presence and prevalence of menstrual blood with other tissues of the body that also have mesenchymal cells, the same PubMed search was made with the following terms, as substitutions to “menstrual blood”: “bone marrow; umbilical cord; umbilical cord blood; umbilical cord vein; Wharton jelly; adipose; placenta; dental pulp; endometrium, and amniotic fluid”. The search terms were: “2008” (Date–Publication): “2020” (Date–Publication) AND human (Title/Abstract) OR mesenchymal (Title/Abstract) OR stromal (Title/Abstract) OR stem cell (Title/Abstract) AND the name of the cell’s origin (Title/Abstract).

As for the social attention analysis, we have used the DOIs, or PubMed ID of the publications sampled to track them on Twitter using the free API from Altmetric.com data provider. Altmetric.com is a data provider of social attention of science literature online. The AAS (altmetric attention score) measures how scientific publications (as articles, book chapters, preprints etc) were mentioned on online platforms such as blogs, news, Wikipedia, Twitter, and Facebook.

To measure the impact of science on society, altmetrics emerge as relevant complementary metrics to scholarly literature on online platforms (Priem et al., 2010). It is a faster and more diverse way to measure the impact of science than the traditional metrics. Yet, the social attention that science outputs receive on social media may differ from that of scholarly interest (Banshal et al., 2018).

We have used the DOIs or PubMed ID to track the 171 articles on Twitter using the free application programming interface (API) from Altmetric.com data provider. Twitter has become one of the main social medias to track social attention scores (such as the Altmetric Attention Score, AAS), it is widely used among scholars (Noorden, 2014; Haustein, 2018) and it gets better coverage within Altmetric.com agregator (Karmakar et al., 2021).

We have manually collected 210 tweets (59.7%) mentioning 26 articles (15.2%) that got an AAS higher than 4.1 from a total of 352 tweets, since Altmetric.com provides only a limited free sample of tweets per paper. The tweets were then categorized by geolocation, profile gender (female, male, group, other or non-identified), profile type (science related, lay person, news, bot, non-identified) type of tweet (comment, retweet, or title and link of the paper), and tone of the comment (positive, negative, or neutral). The tone was considered neutral when it was not for or against the use of mbMSC as most retweets or titles followed by the paper link; positive when optimistic, enthusiastic, or in favor of the use of mbMSC, like sharing its benefits, treatments, or positive results in research; and negative when comments were demeaning, sarcastic, or sharing negative results or limitations in research.

## Results

### Research with menstrual blood cells is minoritarian and mostly conducted by women

Table 1 shows the number of articles found for each of the tissues menstrual blood, bone marrow, umbilical cord, adipose, placenta, dental pulp, endometrium, and amniotic fluid. We considered endometrial cells as different from menstrual blood cells because they are usually obtained by invasive procedures such as hysterectomy, which might result in a different type of cell population. But since both endometrial and menstrual blood cells come from the uterus, we analyzed a sample of 45 papers mentioning endometrial cells (the 15 first, 15 last, and 15 in the middle of search results) to assure that they were not talking about menstrual blood cells when they mentioned endometrium. We read the abstracts and materials and methods of these papers, and only 2 of the 45 showed this ambiguity.

**TABLE 1:** Menstrual blood stromal/stem cells (mbMSCs) represent 0.25% of studies published at PubMed (2008–2020).

Tissue	Number of results	Percentage (%)
menstrual blood	229	0.25
bone marrow	43355	47.71
umbilical cord total	11013	12.12
adipose	20131	22.16
placenta	7721	8.50
dental pulp	3179	3.50
endometrium	3741	4.12
amniotic fluid	1488	1.64
Total	90857	100.00

The comparative analysis shows an extremely low prevalence of publications about menstrual blood mesenchymal stromal/stem cells (0.25% of the research results for the period 2008–2020), in comparison with other tissues, such as bone marrow, umbilical cord, adipose, placenta, dental pulp, endometrium, and amniotic fluid.

Among the 171 articles published about mbMSCs selected from the initial 229, we have noticed a prevalence of women as first authors and men as last authors. We classified as “null” the authors whose gender identity we could not infer. Table 2 shows the numbers for the three outcomes considered (female, male, and null) for the first and last author’s.

**TABLE 2:** Gender of first and last authors of studies about mbMSCs.

	First author	Last author
Female	91	56
Male	69	109
Null	11	6

**FIGURE 2:**
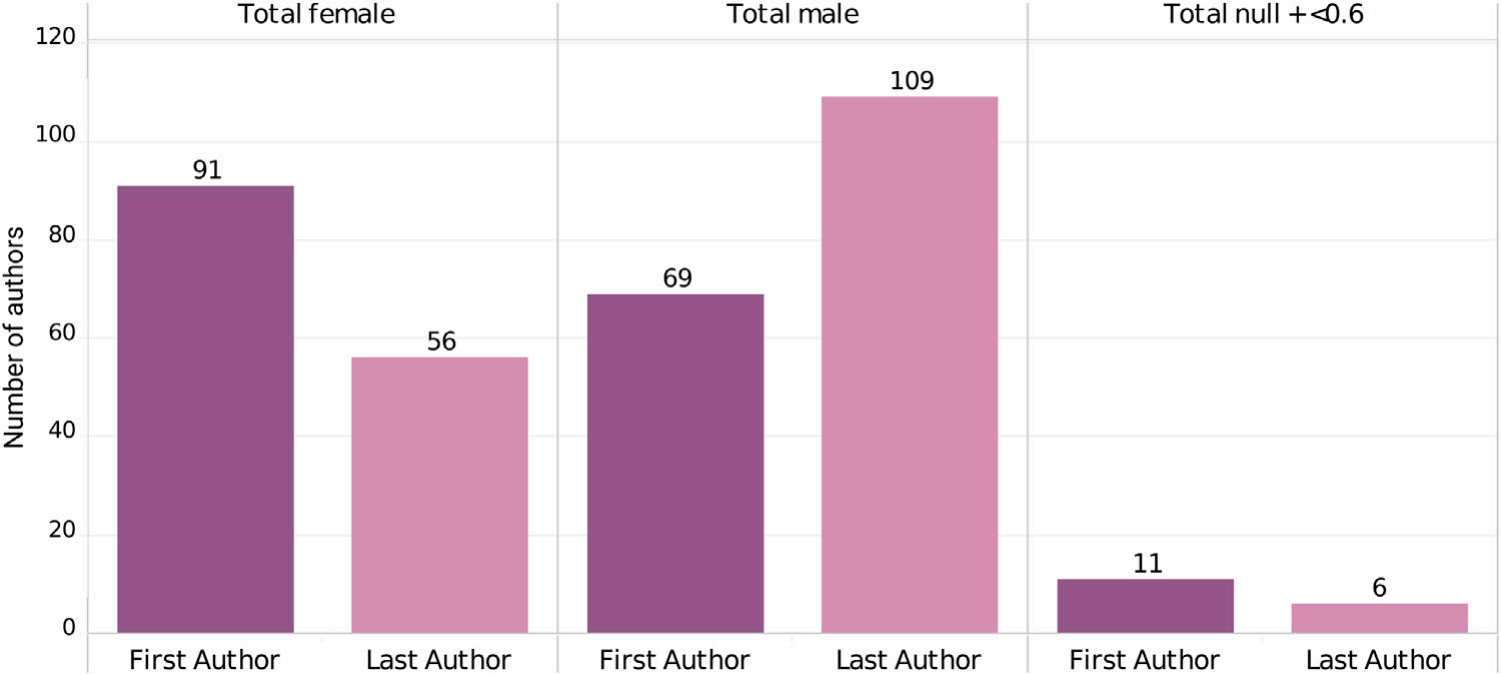
Gender of first and last authors of mbMSC publications.

Table 2 and Figure 2 show 53.2% (91) of first authors were women and 40.35% [69] were men, while men represented 63.74% [109] of last authors and women, 32.74% [56]. The non-identified first and last authors represent 6.43% [11] and 3.5% [6] of the studies, respectively. We established the statistical significance of these results regarding gender identity of the authors of mbMSC publications using a chi-square test (*p* = 0.00008303). A *p* value <0.05 is considered statistically significant.

**FIGURE 3:**
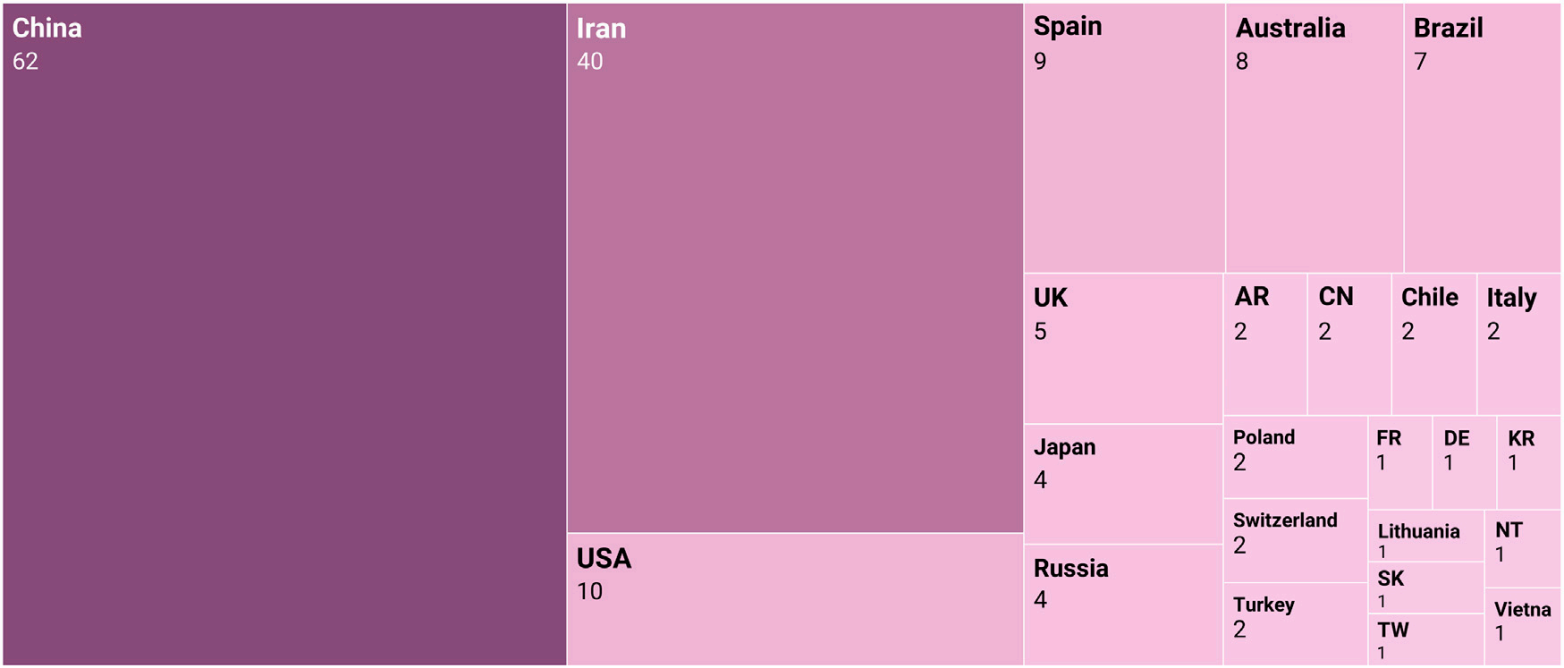
Country of the institution of origin of the last author.

Chinese scholars were the main authors (Figure 3), which represent 62 of the 171 articles analyzed (36.25%). The other authors come from institutions located mostly outside of Europe and the United States: Iran (40, 23.39%), Australia (8, 4.67%), and Brazil (7, 4.09%). The United States has 10 publications, but they come from private research centers (2, 1.17%), and from the University of South Florida (7, 4.09%) and Emory University (1, 0.58%). Spain (Benjamin, 2013) and the United Kingdom (Haraway, 1997) represent 8.19% of the articles published.

**FIGURE 4:**
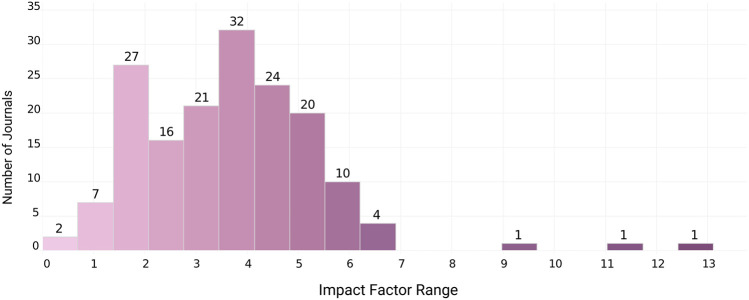
Impact factors of journals that published mbMSCs papers.

Figure 4 shows that the Impact Factors attributed to the journals that published the papers involving menstrual blood cells varied mostly between 1 and 5, suggesting that these publications tend to appear in journals with low/medium impact. Most journals (45.03%) have an impact factor between 3 and 4.99.

**FIGURE 5:**
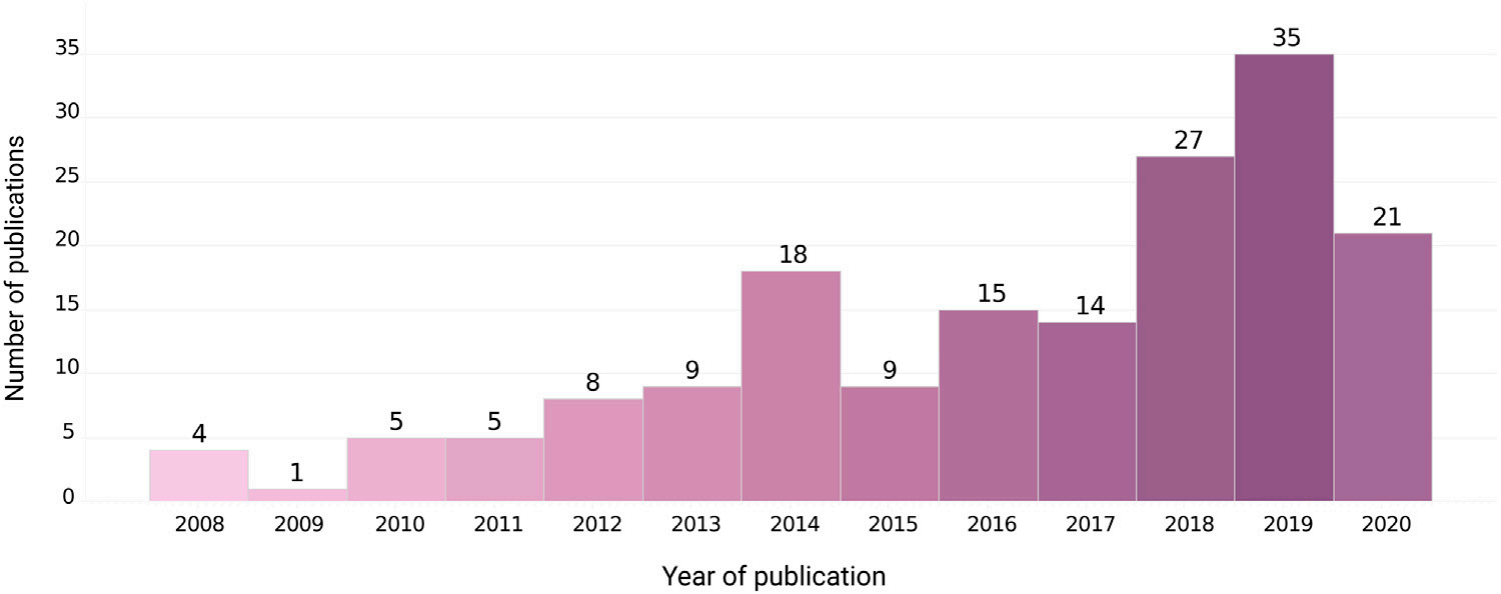
Number of publications with mbMSCs per year.

Figure 5 shows a trend of increasing number of publications, with most papers (83, 48.53%) published since 2018, indicating a growing potential for research involving menstrual blood mesenchymal stromal/stem cells, despite its still small participation among the most representative tissues in scientific research and medical therapies with mesenchymal cells.

### Social attention of menstrual blood mesenchymal stromal/stem cells on twitter

Within our sample, 40.9% of the papers received no altmetric attention score (AAS) and only 5.8% had no Tweets which is less than observed in another study (Karmakar et al., 2021), that registered approximately 50%. This result indicates social interest in mbMSC. Yet, the average AAS was 3.0 per paper, and only 8.2% got an attention score higher than 10, all of which published in open or free access papers, an advantage already reported on altmetrics research (Holmberg et al., 2020).

The Tweet analyses showed that 35.5% of them were comments, which are the best type of engagement when compared to retweets (32.7%) or tweets including the paper title and link (31.8%). Most comments were positive about mbMSC (77.6%), neutral (20.8%), or negative (11.7%), like what stem cell tweet analysis showed (Kamenova et al., 2014; Robillard et al., 2015), and most of them were from lay people. Women with Twitter profiles related to civil rights or feminism made most of the positive comments (36.4%), followed by groups (15.6%)—many of which were surprisingly related to investments or stock market (8.1% among all tweets) –, and men (13%)—mostly scientists. Groups (news feed, labs, institutions, bots, for example) published most of the tweets (38.1%), but they were mainly retweets or just sharing paper titles and links. Therefore, these tweets’ goal is probably to disseminate new papers. Men made all negative comments (6 profiles mainly made by non-scientists), mostly questioning the plausibility and sterility of using these cells [6 comments were related to the article “The Potential of Menstrual Blood-Derived Stem Cells in Differentiation to Epidermal Lineage: A Preliminary Report” (Supplementary Table S1)]. But men also shared some positive (13%) or neutral (7.8%) comments.

Around 61.9% of the tweets were geolocated, a value close to what Robillard et al. (Robillard et al., 2015) found in studies about stem cells (from 63% to 65%). The most active countries were the United States (37.7%), Australia (7.7%), the United Kingdom (6.9%), Japan (6.1%), and Mexico (6.1%), which were different countries from the ones our results indicated as more engaged with mbMSC research.

According to the profile of tweet authorship, most of them (33.8%) were science related (scientists, professors, PhD students, doctor, institutions, journals, and so on), but with intense exchange with lay people (25.7%), investment groups (8%), news feeds (6.2%), bots (5.2%), and others (9%).

## Discussion

### Menstrual blood derived mesenchymal stem cells: State of the art

Studies published using mbMSCs or its products indicate a promising therapeutic potential for several disease models, showing significant benefits *in vitro* and *in vivo*. These cells have already been transplanted intracerebrally and intravenously in a rat model of ischemic stroke and reduced behavioral and histological disorders (Borlongan et al., 2010). In a mouse liver fibrosis model, mbMSC migrated to the injury site, improved liver function, inhibited activated hepatic stellate cells and reduced collagen deposition (Chen et al., 2017). The therapeutic capacity of mbMSC has also been tested in a pulmonary fibrosis mouse model. In this study, cells migrated to the lung and improved its structure, reducing collagen deposition and inflammatory response (Chen et al., 2020). The mbMSC also improved mouse embryonic development *in vitro*, until the blastocyst stage (Gonçalves et al., 2020). Additionally, mbMSC showed good results for *in vivo* models of myocardial infarction (Hida et al., 2008; Jiang et al., 2013), acute liver injury (Lu et al., 2016), spinal cord injury (Wu et al., 2018), duchenne muscular dystrophy (Cui et al., 2007), limb ischemia (Murphy et al., 2008), wound healing (Cuenca et al., 2018; Dalirfardouei et al., 2019), and female reproductive system disorders (Lai et al., 2015; Bu et al., 2016; Tan et al., 2016; Wang et al., 2017; Critchley et al., 2020).

However, despite these positive results, the accessibility of the cell, and its abundant availability, obtained by painless and non-invasive methods, mbMSCs are not among the most studied sources in non-clinical and clinical studies. Until the year of 2020, only three clinical trials were registered in the clinicaltrials.gov database using mbMSCs (NCT01496339, NCT01483248, NCT01558908). Given “the past deficit of attention to female reproductive health and biology” (Critchley et al., 2020) and the absence of technical explanations for that small number, we would like to suggest a gender bias concerning the choices of MSCs sources. In other words, menstrual blood’s relation to gender, as a “feminine” bodily tissue, could explain mbMSC’s lack of expressivity in MSC research.

### Women in benchwork, men as lab chiefs

In Biological Sciences, first authors are usually researchers directly involved with benchwork and with most of the work of writing the paper and organizing the results, while last authors tend to be senior academics, the principal investigators, and tenured professors. Women are underrepresented among last authors, compared to men (West et al., 2013), who figure as most researchers and laboratory supervisors. Our results found that men are more prevalent as last authors, confirming the tendency of having less women among senior researchers, and occupying the higher hierarchical academic positions (Allagnat et al., 2017; Kleijn et al., 2020).

Our results also showed that menstrual blood research is mostly conducted by women scientists, which is different from what other reports on gender and science have demonstrated. As West (West et al., 2013) argues, for example, men usually predominate as first authors in most fields. This result suggests women may be more willing to work with menstrual blood than men. And, as our qualitative research has shown, in contexts in which access to cells and research materials is restricted, women scientists often provide the bodily material themselves, and they tend to have a greater ease to work with menstrual blood, despite jokes and demonstrations of disgust from other scientists (Manica et al., 2018). Concerns found in social media in commentaries made by men about the “sterility” of menstrual blood as a source for cells also confirm a well-known association between menstruation and dirt, impurity, and pollution in western culture (Buckley and Gottlieb, 1988; Bobel et al., 2020).

### Publications from the margins

Europe and the US have produced most of the research about stem cells between 2000–2010 (Karpagam et al., 2012). Our results show that the papers on menstrual blood cells were mainly published by Chinese scholars, situated in Chinese universities. Other research centers are in countries (Iran, Australia, and Brazil) less present among those that concentrate the production on stem cells and in mesenchymal stem cells (Karpagam et al., 2012; Lin and Ho, 2015). The prevalent institutions in the field are Harvard University, University of California, Johns Hopkins, and Stanford (Lin and Ho, 2015), but none of them figure among the American institutions that published results with mbMSCs. This suggests that menstrual blood is unprivileged by the most renowned universities, and that the institutions that have been studying the potentials of mbMSC are less traditional in the stem cell research field.

Analysis of the journals’ impact factors include menstrual blood research within the array of a low/regular-impact scientific production, which confirms mbMSC’s absence in higher impact scientific journals (Chen et al., 2014; Lin and Ho, 2015). Most papers were published in the last 3 years, which demonstrates a growing potential for menstrual blood mesenchymal stromal/stem cells research, despite the obstacles to its inclusion among the most representative tissues in scientific research and medical therapies with mesenchymal cells.

### Menstrual blood cells research on twitter: Potentially interesting

Studies have shown that women’s scientific work is less cited and gets less visibility on social media than men’s (Vásárhelyi et al., 2021). Social media is an important space for gaining credibility, visibility, and success, therefore it is relevant to strengthen the presence of women in science. Vásárhelyi and colleagues (Vásárhelyi et al., 2021) have concluded that even in fields where women are better represented, such as medical sciences, their online presence on social media remains lower (39%) than men’s (61%).

Our results have shown that mbMSC papers have raised social attention on Twitter, and as communication on social media has grown worldwide and became a key space to monitor social interaction with information, it is a key space to improve visibility and dialogue about those cells and the work of women in science.

Thomlinson (Thomlinson, 2021) has analyzed 220 menstrual memes on Instagram and although she has found a reinforcement of stigma, she argues memes have a great potential to normalize menstruation and strengthen menstrual activism. According to the author, “it calls for scholars within critical menstruation studies to revaluate the role of popular culture in shaping societal attitudes towards menstruation.“. Likewise, Twitter has shown a positive engagement around menstrual blood cells, but there is still space to strengthen visibility among influencers and scientists for the high quality of menstrual blood cells in research.

Our analysis has confirmed that women are more engaged with the social debate related to menstrual blood stem cells, and men, despite also contributing with positive comments, are responsible for all the negative tweet comments. This could suggest that women researchers of mbMSC should be more active on Twitter to reinforce the visibility of their work and of their research field.

We found that scientists, institutions, and journals responsible for merely sharing paper titles and links could try to engage with the audience in online debates and interactions. Tweets about mbMSC papers tend to get social attention and interest, and therefore have great potential to communicate scientific results broadly, as suggested by the good number of tweets from lay people, including feminist and profiles related to investment or stock market.

Contrary to our hypothesis, tweets reveal no bias against the use of mbMSC, since positive comments were almost six times more frequent than negative comments.

Despite mbMSCs attending all the required expectancies regarding the potential of mesenchymal stromal/stem cells, some work still needs to be done for menstrual blood to occupy its proper place in the Universe of mesenchymal cell research, overcoming “the established infrastructure that relies on bone marrow, adipose tissue, and other sources” (Critchley et al., 2020).

### Limitations of the study

Pubmed database presented instabilities and changes in the online platform during our research. Some papers appeared simultaneously in 2 years, especially when approved and published in different years. We have identified and excluded them from the table.

The set of articles considered, selected through Pubmed, has the limitation of only covering articles indexed in this platform, leaving a number of potential papers indexed in other search bases out. The keywords chosen were intended to include publications in the area of mesenchymal cells, and this also leaves out other possible applications of menstrual blood, as well as other approaches to menstruation in the biomedical sciences.

Inferring gender by names disregards many important variables, like gender transition, non-binary or androgynous names or physical appearances. Gender cannot often be inferred for Chinese names in the Roman alphabet. Nonetheless, we have considered it was still important to look for differences between what could be considered as men and women among first and last authors, using the software genderize. io to help us infer gender in those cases, and others. We did not consider the other co-authors beyond the first and last author, and possibly if we had considered the entire team of coauthors the results might be diverse.

There are other bases and software for evaluating the social impact of scientific production, besides Altmetrics, that were not considered in the analysis, as Plum X. We also have analyzed tweets but not posts on Facebook or other social media, so the conversation about menstrual blood cells might be different in other platforms. As for the analysis with altmetrics.com, we know the tweets analyzed cannot be generalized. Yet, our sample corresponds to 55.5% of the tweets received by the selected mbMSCs article and considers studies with more tweets and higher AAS, to select the most representative expressions about the studies.

## Conclusion

In this paper, we analyze the publication of papers on menstrual blood stromal mesenchymal cells in the Pubmed database between 2008 and 2020. Several papers in this period point to the excellence of menstrual blood as a basis for cell therapy, regenerative medicine and bioengineering. However, our research shows that the representativeness of menstrual blood research in the Universe of mesenchymal cells is small (0.25%). Considering the state-of-the-art literature we have retrieved in the text, and the easy access to menstrual blood cells, we conclude that there would be no technical explanation for such an expressive preference for other tissues, which require much more invasive procedures to be obtained and have a lower relative availability of cells. The reason is linked to the fact that it is a tissue marked by social constraints and gender bias.

Looking at the profile of scientists working with menstrual blood, we observe that they are mostly women, from non-hegemonic universities and research centers (in China, Iran and Australia). The finding of women as first authors differs from that seen in other papers that examine the relationship between gender and authorship, and in which most first authors are men. The last authors, on the other hand, are mostly men, as is common in other fields, in which men also occupy the majority of leadership and decision-making positions. Publications on menstrual blood cells tend to appear in low/medium impact journals. The profiles of the authors, universities and journals that publish the results of this research allow us to say that the use of menstrual blood in the Universe of mesenchymal cell research is incipient and is being carried out by minority groups, without significant space in the central research agenda. The receptivity of menstrual blood research in Twitter, on the other hand, is generally good and might be exploited as an important means to make menstrual blood cells more visible.

## Data Availability

The datasets presented in this study can be found in online repositories. The names of the repository/repositories and accession number(s) can be found below: Manica Daniela Tonelli, 2022. “Table with Pubmed research output. ods,” Corpo, gênero e tecnociências: https://doi.org/10.25824/redu/BBHTSY, Repositório de Dados de Pesquisa da Unicamp, V1.
