# Supplementary material for: Gender bias and menstrual blood in stem cell research: A review of pubmed articles (2008–2020)
Source: Front Genet. 2022 Dec 1;13:957164. doi: 10.3389/fgene.2022.957164 (PMC9754585; doi:10.3389/fgene.2022.957164)
Supplement: Supplementary file 1 [file DataSheet1.PDF]

First author	Sex	Sex-Software	Country (1st author)	Last author	Sex	Sex-Softw	Country (last author)	Publishing date	Journal - title	Impact Factor	DOI
Caroline Gargett	F		Australia	James Deane	M		Australia	2015	Human Reproduction Update	11.194	10.1093/humupd/dmv051
Maria Carolina Rodrigues	F		Brazil	Cesar Borlongan	M		USA	2016	Biobanking and Cryopreservation of Stem Cells (E	book	10.1007/978-3-319-45457-3
Connor Stonesifer	M		USA	Cesar Borlongan	M		USA	2017	Progress in Neurobiology	12.716	10.1016/j.pneurobio.2017.07.004
Lu Chen		F 0.57	China	Charlie Xiang	M		China	2017	Stem Cell Research & Therapy	5.278	10.1186/s13287-016-0453-6
Cesar Borlongan	M		USA	Paul R Sanberg	M		USA	2010	Stem Cells And Development	4.922	10.1089/scd.2009.0340
Fahimeh Tabatabaei	F		Iran	Jafah Ai	M		Iran	2017	Regenerative Medicine	2.664	10.2217/rme-2017-0029
Ilona Uzielienė	F		Lithuania	Eiva Bernotiene	F		Lithuania	2018	Stem Cells International	3.937	10.1155/2018/5748126
Huaijuan Ren		null	China	Yantian Chen		F 1.0	China	2015	Stem Cells International	4.616	10.1155/2016/3516574
Yanli Liu		F 0.87	China	Juntang Lin		M 1.0	China	2017	Journal of Cellular and Molecular Medicine	4.439	10.1111/jcmm.13437
Bingyu Xiang		F 0.64	China	Charlie Xiang	M		China	2017	International Journal of Molecular Sciences	3.962	10.3390/ijms18040689
David Eve	M		USA	Krystyna Domanska	F		Poland	2018	Human Neural Stem Cells (Book)	book	10.1007/978-3-319-93485-3
Martin A Ivarsson	M		UK	Niklas K. Bjorkstrom	M		UK	2018	Mucosal Immunology	6.859	10.1038/mi.2016.50
Hossein Eyni	M		Iran	Masoud Soleimani	M		Iran	2017	Journal of Biomaterial Applications	2.264	10.1177/0885328217723179
Dongmei Lai		F 0.98	China	Charlie Xiang	M		China	2015	Journal of Translational Medicine	4.222	10.1186/s12967-015-0516-y
Haining Lv		M 1.0	China	Huidong Jia	M		UK	2018	Stem Cell Research & Therapy	4.777	10.1186/s13287-018-1067-y
Mehdi Aleahmad	M		Iran	Siwen Zhang	M		Iran	2018	Avicenna Journal of Medical Biotechnology	1.55	PMID: 30090214
Xiao-Jun Wang	M		China	Charlie Xiang	M		China	2017	Oncotarget	4.849	10.18632/oncotarget.17621
Saeed Farzamfar	M		Iran	Majid Salehi	M		Iran	2017	Molecular Biology Reports	2.178	10.1007/s11033-017-4124-1
Zahra Rajabi	F		Iran	Amir-Hassan Zarnar	M		Iran	2018	Reproductive Biology	2.043	10.1016/j.repbio.2018.02.001
Karina Asensi	F		Brazil	Regina Goldenberg	F		Brazil	2014	Journal of Cellular and Molecular Medicine	4.605	10.1111/jcmm.12226
Razieh Dalirfardouei	F		Iran	Elahe Mahdipour	F		Iran	2018	Tissue and Cell	1.553	10.1016/j.tice.2018.09.010
Yanling Zhang	F		China	Songying Zhang	F		China	2016	Reproduction Research	3.514	10.1530/REP-16-0286
Nikoo Shojanoori	F		Iran	Amir-Hassan Zarnar	M		Iran	2012	The Journal of Obstetrics and Gynaecology Resea	1.128	10.1111/j.1447-0756.2011.01800.x
Shulan Zhang	F		China	Jingwei Tan	F		China	2018	Stem Cell Research & Therapy	4.777	10.1186/s13287-018-0795-3
Fereshteh Azedi	F		Iran	Shaghayegh Arastel	F		Iran	2017	Molecular Biology Reports	2.178	10.1007/s11033-016-4095-7
Dongmei Lai		F 0.98	China	Charlie Xiang	M		China	2016	Acta Biochimica et Biophysica Sinica	2.148	10.1093/abbs/gmw090
Yuliang Sun		M 0.93	China	Juntang Lin		M 1.0	China	2019	Biology Open	2.026	10.1242/bio.038885
Paul R. Sanberg	M		USA	Cesar V Borlongan	M		USA	2011	Cell Transplantation	4.116	10.3727/096368910X532855
Daniela Ulrich	F		Australia	Caroline Gargett	F		Australia	2013	Expert Opinion Biological Therapy	4.120	10.1517/14712598.2013.826187
Kana Sugawara		F 0.8	Japan	Akihiro Umezawa	M		Japan	2014	Scientific Reports	5.990	10.1038/srep04599
Mahmood Bozorgmerh	M		Iran	Amir-Hassan Zarnar	M		Iran	2014	Immunology Letters	2.868	10.1016/j.imlet.2014.10.005
Mina Fathi-Kazerooni	F		Iran	Somaieh Kazemneja	F		Iran	2017	Cytotherapy	4.017	10.1016/j.jcyt.2017.08.022
Davood Mehrabani	M		Iran	Farnaz Ghobadi	F		Iran	2016	Iranian Journal of Medical Sciences	0.94	PMID: 26989284
Filippo Rossignoli	M		Italy	Massimo Dominici	M		Italy	2013	BioMed Research International	3.276	10.1155/2013/901821
Shohreh Nikoo	F		Iran	Amir-Hassan Zarnar	M		Iran	2014	Molecular Human Reproduction	4.209	10.1093/molehr/gau044
Frederico Allison-Silva	M		Brazil	Adriane Todeschini	F		Brazil	2014	Glycobiology	3.595	10.1093/glycob/cwu012
Yanping Xu		F 0.7	China	Jichun Tan	M		China	2015	International Journal of Clinical and Experimental	1.316	PMID: 26885178
Xue Du	F		China	Jia Bei	F		UK	2016	Stem Cells International	3.756	10.1155/2016/3573846
R Moreno			Spain	Rafael Alemany	M		Spain	2017	Stem Cells International	3.959	10.1155/2017/3615729
Te Liu	M		China	Chuan Chen		M 0.86	China	2014	Stem Cells International	3.582	10.1089/scd.2013.0371
Marina V. Kovina	F		Russia	Alexey V. Lyubdup	M		Russia	2018	Cytotherapy	4.120	10.1016/j.jcyt.2017.12.012
Danubia Silva dos Santos	F		Brazil	Regina Goldenberg	F		Brazil	2014	Cell Medicine		10.3727/215517914X679265
Saeedeh Darzi	F		Iran	Somaieh Kazemneja	F		Iran	2012	Tissue Engineering	4.946	10.1089/ten.tea.2011.0386
Irina Kozhkharova	F		Russia	Nikolay Nikolsky	M		Russia	2017	International Journal of Hematology	2.152	10.1007/s12185-017-2346-6
Lijun Chen		F 0.51	China	Charlie Xiang	M		China	2016	Stem Cells Translational Medicine	4.672	10.5966/sctm.2015-0265
Natalia Russo	F		Italy	Andrea Riccardo Ge	M		Italy	2012	Gynecological Endocrinology	1.607	10.3109/09513590.2011.633667
Rafael Moreno	M		Spain	Ramon Alemany	M		Spain	2018	Molecular Cancer Therapeutics	5.151	10.1158/1535-7163.MCT-18-0431
Deivid de Carvalho Rodri	M		Brazil	Turán Péter Ürmény	M		Brazil	2012	Cell Transplantation	4.810	10.3727/096368912X653048
Saeed Farzamfar	M		Iran	Mehdi Aleahmad	M		Iran	2018	Biomedical Engineering Letters	1,2	10.1007/s13534-018-0084-1
Naoko Hida	F		Japan	Akihiro Umezawa	M		Japan	2008	Stem Cells	9.162	10.1634/stemcells.2007-0826
Yongjia Zhao		M 0.71	Japan	Charlie Xiang	M		Japan	2018	Frontiers in Molecular Neuroscience	3.814	10.3389/fnmol.2018.00140
Zhaocai Zhang		M 1.0	China	Hong Yo		M 0.65	China	2013	International Journal of Cardiology	1.882	10.1016/j.ijcard.2013.03.126

Patricia Luz-Crawford	F		Chile	Maroun Khoury	M		Chile	2015	Stem Cells	6.585	10.1002/stem.2244
Dah-Ching Ding	F		Taiwan	Shinn-Zong Lin	M		Taiwan	2011	Cell Transplatation	4.116	10.3727/096368910X
Jinyang Chen		M 0.69	China	Charlie Xiang	M		China	2015	International Journal of Clinical and Experimental	1.825	PMID: 26823782
Jichun Tan	M		China	Lin Kong		F 0.63	China	2016	Human Reproducction	5.020	10.1093/humrep/dew235
Julie Allickson	F		USA	Paul R. Sanberg	M		USA	2011	Open Stem Cell Journal		10.2174/1876893801103010004
Haiyan Zhu		F 0.9	China	Songying Zhang	F		China	2018	Reproductive Biology	2.043	10.1016/j.repbio.2018.06.003
Birol Ay	M		Turkey	Halime Kenar	F		Turkey	2016	Journal of Biomedical Materials Research Part A	3.607	10.1002/jbm.a.35948
Jian Lin		M 0.9	China	Charlie Xiang	M		China	2011	Journal of Zhejiang University	1.482	10.1631/jzus.B1100015
Shanti Gurung	F		Australia	Caroline E. Garget	F		Australia	2015	Seminars in Reproductive Medicine	2.776	10.1055/s-0035-1558405
Shaghayegh Arasteh	F		Iran	**Somaieh Kazemne**	F		Iran	2018	Stem Cell Nanotechnology (book)	book	10.1007/7651_2018_193
Maroun Khoury	M		Chile	Fernando E. Figuero	M		Chile	2014	Frontier in Immunology	4.994	10.3389/fimmu.2014.00205
Shixia Bu		F 1.0	China	Dongmei Lai		F 0.98	China	2016	Scientific Reports	4.738	10.1038/srep37019
Ozge Karadas	F		Turkey	Vasif Hasirci	M		Turkey	2012	Journal of Tissue Engengering and Regenerative	2.989	10.1002/term.1555
Somaieh Kazemnejad	F		Iran	Saghari S	M		Iran	2013	Journal of Stem Cells and Regenerative Medicine	0.22	10.46582/jsrm.0901004
Caroline E. Gargett	F		Australia	Hirotaka Masuda	M		Australia	2010	Molecular Human Reproducction	3.660	10.1093/molehr/gaq061
Sheng-Xia Zheng		M 1.0	China	Yu-Sheng Liu	M		China	2018	International Journal of Molecular Medicine	3.024	10.3892/ijmm.2018.3415
Xuqi Hu		F 1.0	China	Huazi Xu		M 0.64	China	2014	Spine Journal	2.445	10.1097/BRS.0000000000000261
Hossein Faramarzi	M		Iran	Reza Shirazi	M		Iran	2016	World Journal of Plastic Surgery		PMID: 27308237
Qinfeng Wu		M 1.0	China	Chuanming Dong		M 1.0	China	2018	Cell Death and Disease	6.083	10.1038/s41419-018-0847-8
Julie Allickson	F		USA	Charlie Xiang	M		China	2012	Journal of Zhejiang University - Science B	1.456	10.1631/jzus.B1200062
Yijing Zheng		F 0.88	China	Jianjun Hong		M 0.97	China	2017	Experimental and Therapeutic Medicine	1.551	10.3892/etm.2017.4383
Manijeh Khanmohammad	F		Iran	Somaieh Kazemneja	F		Iran	2012	International Journal of Hematology	2.106	10.1007/s12185-012-1067-0
Xiao- Zhou Mou	(fotc	M 0.75	China	Charlie Xiang	M		China	2013	Journal of Zhejiang University - Science B	1.733	10.1631/jzus.B1300081
Stanimir Kyurkchiev	M		Bulgaria	Rumen Dimitrov	M		Bulgaria	2010	Reproducttive BioMedicine Online	2.869	10.1016/j.rbmo.2009.12.011
Maryam Fard	F		Iran	Reza Shirazi	M		Iran	2018	Molecular Biotechnology	1.811	10.1007/s12033-017-0049-0
Haitao Ren		M 0.94	China	Ruolang Pan		null	China	2018	Stem Cells International	3.937	10.1155/2018/7873625
Xiaoxi Xu		F 0.6	Canada	Hao Wang	M		Canada	2016	Stem Cells Translational Medicine	4.672	10.5966/sctm.2016-0206
Yanli Liu		F 0.87	China	Juntang Lin		F 0.98	China	2018	Stem Cells International	3.937	10.1155/2018/3250379
Naoki Tajiri	M		USA	Yuji Kaneko	M		USA	2014	International Journal of Molecular Sciences	3.385	10.3390/ijms150915225
Amit N. Patel	M		USA	Julie allickson	F		USA	2008	Cell Transplantation	4.356	10.3727/096368908784153922
Manijeh Khanmohammad	F		Iran	Somaieh Kazemneja	F		Iran	2014	Cell Proliferation	3.734	10.1111/cpr.12133
Ana Laura Alfano	F		Argentina	Veronica Lopez	F		Argentina	2017	Molecular Therapy Oncolutics	3.915	10.1016/j.omto.2017.06.002
Alisa P. Domnina	F		Russia	Nikolay N. Nikolsky		M 0.99	Russia	2016	Experimental and Therapeutic Medicne	1.490	10.3892/etm.2016.3671
Bruna R. Sousa	F		Brazil	Rodrigo R. Resende	M		Brazil	2013	Cytometry Part A	3.421	10.1002/cyto.a.22402
Sayeh Khanjani	F		Iran	Somaieh Kazemneja	F		Iran	2014	PLOS ONE	3.778	10.1371/journal.pone.0086075
Xiaohan Wang		F 0.69	China	Cuifang Hao		null	China	2018	Journal of Cellular Biochemistry	3.448	10.1002/jcb.28014
JIn-Yang Chen		M 0.69	China	Charlie Xiang	M		China	2015	Asian Pacific Journal of Tropical Medicine	1.630	10.1016/j.apjtm.2015.07.022
Yang Li		M 0.64	China	Jichun Tan	M		China	2013	Stem Cells and Development	4.710	10.1089/scd.2012.0428
Pham Van Phuc	M		Vietnam	Phan Kim Ngoc	M		Vietnam	2011	In Vitro Cellular & Developmental Biology - Anima	1.665	10.1007/s11626-011-9399-2
Federica Marino	F		Spain	Beatriz Macías- Gar	F		Spain	2018	Reproduction in Domestic Animals	1.614	10.1111/rda.13314
Somaieh Kazemnejad	F		Iran	Kamran Alimoghada	M		Iran	2012	The International Journal of Artificial Organs	1.961	10.5301/ijao.5000019
Sayeh Khanjani	F		Iran	Somaieh Kazemneja	F		Iran	2015	Journal of Tissue Engineering and Regenerative M	3.211	10.1002/term.1715
Xiaoxing Wu		M 0.56	China	Charlie Xiang	M		China	2014	Stem Cells and Development	4.349	10.1089/scd.2013.0390
Marjan D. Manshadi	F		Iran	Mehdi Abbasi	M		Iran	2018	Microscopy Research and Technique	1.355	10.1002/jemt.23120
Javad Verdi	M		UK	Alexander M. Seifali	M		UK	2014	Journal of Biological Engeneering	3.143	10.1186/1754-1611-8-20
Fengyi Gou		M 0.89	China	Xue Du	F		China	2019	Reproductive Biology and Endocrionology	3.498	10.1186/s12958-019-0499-2
Zhi Jaing		M 0.75	China	Jian-an Wang	M		China	2013	Journal of Cellular and Molecular Medicine	4.258	10.1111/jcmm.12100
Fereshteh Azedi	F		Iran	Niknam Lakpour	M		Iran	2014	Cell Biollogy International	2.160	10.1002/cbin.10245
Yongcheng Lv			China	Hao Wang		M 0.89	China	2014	Journal of Translational Medicine	4.630	10.1186/s12967-014-0344-5
Amit N. Patel	M		USA	Francisco Silva	M		Argentina	2008	Regenerative Medicine	2.950	10.1016/j.hfc.2014.12.006
Maryam Rahimi	F		Iran	Somaieh Kazemneja	F		Iran	2014	Molecular Biotechnology	2.215	10.1007/s12033-014-9795-4
Shanzheng Lu			China	Hao Wang		M 0.89	China	2016	Journal of Translational Medicine	4.155	10.1186/s12967-016-1051-1
Hongyun Huang		M 1.0	China	Paul Sanberg	M		USA	2010	Cell Medicine		10.3727/215517910X516673

James A. Deane	M		Australia	Caroline E. Gargett	F		Australia	2013	Current Opinion in Obstetrics & Gynecology	3.061	10.1097/GCO.0b013e32836024e7
Yukinori Ikegami	M		Japan	Akihiro Umezawa	M		Japan	2010	Artificial Organs	1.654	10.1111/j.1525-1594.2009.00859.x
Jia Hu		F 0.63	China	Bu-Zhen Tan		Null	China	2019	Molecular Medicine Reports	2.085	10.3892/mmr.2018.9744
Maryam Rahimi	F		Iran	Somaieh Kazemneja	F		Iran	2014	Journal of Biomaterial Applications	2.365	10.1177/0885328213519835
Penghui Feng		M 1.0	China	Jichun Tan	M		China	2018	Stem Cell Reviews and Reports	4.924	10.1007/s12015-018-9867-0
Peng Sun		M 0.84	China	Hao Wang		M 0.89	China	2016	Journal of Translational Medicine	4.155	10.1186/s12967-016-0782-3
Yiming Zhao		M 0.82	China	Hao Wang		M 0.89	China	2018	Stem Cells International	3.937	10.1155/2018/3475137
Man-Jing Zhang		F 1.0	China	Kai-Hua Lu		M 0.86	China	2009	Medical Hypotheses	1.700	10.1016/j.mehy.2008.10.021
Xiaoxi Xu		M 0.56	China	Hao Wang		M 0.89	China	2018	Stem Cell Research & Therapy	4.777	10.1186/s13287-018-0874-5
Michael P. Murphy	M		USA	Niel H. Riordan	M		USA	2008	Journal of Translational Medicine	3.361	10.1186/1479-5876-6-45
Xiuhui Chen		F 0.86	China	Meimei Liu		F 0.96	China	2016	International Journal of Molecular Medicine	2.579	10.3892/ijmm.2016.2593
Mohammad-Reza Shokri	M		Iran	Amir-Hassan Zarnar	M		Iran	2019	Scientific Reports	4.149	10.1038/s41598-019-46316-3
Xinxin Zhu	F		China	Lijun Ding		F 0.51	China	2019	Current Stem Cell Research & Therapy	2.614	10.2174/1574888X14666181205120110
Rafael Moreno	M		Spain	Ramon Alemany	M		Spain	2018	Molecular Cancer Therapeutics	5.151	10.1158/1535-7163.MCT-18-0431
Lijun Chen	M Foto	F 0.51	China	Charlie Xiang	M		China	2019	Stem Cell Research & Therapy	5.081	10.1186/s13287-018-1105-9
Yun-xia Zhao	F		China	Shu Lin		F 0.57	Australia	2019	Stem Cell International	3.873	10.1155/2019/9071720
Lijun Chen	M Foto	F 0.51	China	Charlie Xiang	M		China	2019	Stem Cell Research & Therapy	5.081	10.1186/s13287-019-1503-7
Aneta Sciezynska	F		Poland	Jacek Malejczyk	M		Poland	2019	Journal of Clinical Medicine	5.583	10.3390/jcm8091468
Siwen Zhang	M		China	Jichun Tan	M		China	2019	Stem Cell Research & Therapy	5.081	10.1186/s13287-019-1155-7
Qian-Yu Liu		F 0.7	China	Quan-Wen Liu		M 1.0	China	2019	Stem Cell International	3.873	10.1155/2019/9280298
Yi-Chen Wu	F (foto	M 0.6	China	Charlie Xiang	M		China	2019	Stem Cell Research & Therapy	5.081	10.1186/s13287-019-1243-8
Marjan D. Manshadi	F		Iran	Mehdi Abbasi	M		Iran	2018	Microscopy Research and Technique	1.355	10.1002/jemt.23120
Yanli Liu		F 0.87	China	Nan Ma	F		Germany	2019	Cellular and Molecular Life Sciences	6.484	10.1007/s00018-019-03019-2
Parastoo Noory	F		Iran	Mehdi Abbasi	M		Iran	2019	Cellular Reprogramming	1.682	10.1089/cell.2019.0020
Pan-Pan Cen		M 0.62	China	Lan-Juan Li		F 1.0	China	2019	World Journal of Gastroenterology	3.602	10.3748/wjg.v25.i41.6190
Elahe Mahdipour	F		Iran	Nona Sabeti		F 0.95	Iran	2019	Journal of Cellular Physiology	5.546	10.1002/jcp.28631
Yang Guo		M 0.64	China	Charlie Xiang	M		China	2019	Stem Cell and Development	3.145	10.1089/scd.2018.0222
Paula Barlabé	F		Spain	Rafael Moreno	M		Spain	2019	Cancer Gene Therapy	4.472	10.1038/s41417-019-0110-1
Hanna Manley	F		UK	Philip Breedon	M		UK	2019	Journa of Women's Health	2.332	10.1089/jwh.2019.7745
Song Chen		M 0.69	China	Jian Chen		M 0.9	China	2019	In Vitro Cellular & Develoopmental Biology - Anim	1.587	10.1007/s11626-018-0311-1
Han Li		M 0.73	Malaysia	Juntang Lin		M 1.0	China	2019	Frontiers in Molecular Neuroscience	4.161	10.3389/fnmol.2019.00080
Razieh Dalirfardouei	F		Iran	Elahe Mahdipour	F		Iran	2019	Journal of Tissue Enguneering and Regenerative	3.241	10.1002/term.2799
Pavel Deryabin	M		Russia	Aleksandra Borodkir	F		Russia	2019	Cell Cycle	3.699	10.1080/15384101.2019.1593650
Mina Fathi-Kazerooni	F		Iran	Gholamreza Tavoos	M		Iran	2019	Avicenna Journal of Medical Biotechnology	1.22	PMID: 31908739
Andrea Patrizia Salzman	F		Switzerland	Cordula Haas	F		Switzerland	2019	Forensic Science International: Genetics	5.786	10.1016/j.fsigen.2019.102149
Akos Dobay	M		Switzerland	Natasha Arora	F		Switzerland	2019	Forensic Science International: Genetics	5.786	10.1016/j.fsigen.2019.02.010
Ganggang Shi		M 0.94	China	Hao Wang		M 0.89	China	2019	American Journal of Translational Research	3.375	PMID: 31497192
Manijeh Khanmohammad	F		Iran	Somaieh Kazemneja	F		Iran	2019	Tissue Engineering and Regenerative Medicine	3.241	10.1007/s13770-019-00189-9
Zhongrui Yan		M 1.0	China	Xue Du	F		China	2019	Stem Cell Research & Therapy	5.081	10.1186/s13287-018-1101-0
Penghui Feng		M 1.0	China	Jichun Tan		M 0.67	China	2019	Stem Cell Reviews and Reports	5.353	10.1007/s12015-018-9867-0
Shouyu Wang		F 0.5	China	Yiping Hou (M - news)		M 0.53	China	2019	Forensic Science International: Genetics	4.884	10.1016/j.fsigen.2019.01.002
Marine Legrand	F		France	Anais Tondeur	F		France	2019	Journal International de Bioethique et D'ethique d	dossie	10.3917/jibes.304.0019
Mina Fathi-Kazerooni	F		Iran	Gholamreza Tavoosidana		M 0.99	Iran	2019	Biologicals	1.795	10.1016/j.biologicals.2019.02.002
Sook Young Yoon		F 0.92	Korea	autor único		F 0.92	Korea	2019	Clinical and Experimental Reproductive Medicine	1.688	10.5653/cerm.2019.46.1.1
Alireza Ghanavatinejad	M		Iran	Amir-Hassan Zarnar	M		Iran	2020	Journal of Reproductive Immunology	3.535	10.1016/j.jri.2020.103252
Ebrahim Mirzadegan	M		Iran	Somaieh Kazemneja	F		Iran	2020	International Immunopharmacology	3.943	10.1016/j.intimp.2020.106595
Celia Díez López	F		Netherlands	Manfred Kayser	M		Netherlands	2020	Forensic Science International: Genetics	4.117	10.1016/j.fsigen.2020.102280
Paula Barlabé	F		Spain	Rafael Moreno	M		Spain	2020	Cancer Gene Therapy	4.883	10.1038/s41417-019-0110-1
Xin Chen		F 0.52	China	Charlie Xiang	M		China	2020	Stem Cell Research & Therapy	5.985	10.1186/s13287-020-01926-x
Rocio Martínez-Aguilar		F 0.99	Spain	Ana C. Abadía-Molir	F		Spain	2020	Scientific Reports	4.13	10.1038/s41598-020-78423-x
Limei Chen		F 1.0	China	Long Sui		M 0.93	China	2020	American Journal of Translational Research	3.827	PMID:33042399
Shaghayegh Arasteh	F		Iran	Somaieh Kazemneja	F		Iran	2020	Stem Cell Nanotechnology (Book)	book	10.1007/7651_2018_193
Zidong Liu		M 1.0	China	Gengqian Zhang		M (Foto or	China	2020	Electrophoresis Journal	3.062	10.1002/elps.202000053

Mahmood Bozorgmehr	M		Iran	Caroline E. Gargett	F		Australia	2020	Frontiers in Cell and Developmental Biology	5.868	10.3389/fcell.2020.00497
Rosana de Almeida Sant	F		Brazil	Regina Coeli dos Sa	F		Brazil	2020	International Journal of Molecular Sciences	5.542	10.3390/ijms21249563
Sedighe Esmaeilzadeh	F		Iran	Mohammad Ghasen	M		Iran	2020	The Journal of Obstetrics and Gynaecology Resea	1.524	10.1111/jog.14340
Shanti Gurung	F		Australia	Caroline E. Gargett	F		Australia	2020	Journal of Personalized Medicine	1.462	10.3390/jpm10040261
Alicia Sanzhez-Mata	F		Spain	Elena Gonzalez-Mu	F		Spain	2020	iScience	5.080	10.1016/j.xpro.2020.100183
Jiajia Chen		F 0.78	China	Lanjuan Li		F 1.0	China	2020	Engineering	6.495	10.1016/j.eng.2020.02.006
Lidia Lopez-Caraballo	F		Spain	Elena Gonzalez-M	F		Spain	2020	iScience	5.080	10.1016/j.isci.2020.101376
Wang Jin		M 0.67	China	Hao Wang		M 0.89	China	2020	Stem Cells International	4.72	10.1155/2020/4820543
Wenchun Qu	M		USA	Fred P. Sanfilippo	M		USA	2020	Stem Cells Translational Medicine	5.607	10.1002/sctm.20-0146
Marianna Ferreira Gonça	F		Brazil	Regina Coeli dos Sa	F		Brazil	2020	Tissue Engineering	3.312	10.1089/ten.tea.2020.0034
Qi-Yuan Chang		M 0.83	China	Ji-Chun Tan		M 0.67-0.7	China	2020	World Journal of Stem Cells	5.326	10.4252/wjsc.v12.i5.368
Simin Zafardoust		F 0.88	Iran	Afsaneh Mohammadzadeh		F 0.98	Iran	2020	Stem Cell Reviews and Reports	4.813	10.1007/s12015-020-09969-6
Hyun Sok Chung		M 0.63	South Korea	Autor único		M 0.63		2019	Korean Journal of Medical History	0.12	10.13081/kjmh.2019.28.239
